# Fingerprint analysis for the determination of hand origin (right/left) using the axis slant in whorl patterns

**DOI:** 10.1080/20961790.2020.1794362

**Published:** 2020-08-12

**Authors:** Neeti Kapoor, Ashish Badiye, Swati Dubey Mishra

**Affiliations:** a Department of Forensic Science, Government Institute of Forensic Science, Nagpur, Maharashtra, India; b Shri Vaishnav Institute of Forensic Science, Shri Vaishnav Vidyapeeth Vishwavidyalaya, Indore, Madhya Pradesh, India

**Keywords:** Forensic sciences, side determination, whorl slant, fingerprint analysis, crime, hand

## Abstract

Fingerprints are frequently encountered during both civil and criminal investigations. Fingerprints possess numerous characteristics that assist with personal identification. Determining the hand of origin (right or left) for an individual fingerprint would help reduce investigation time and potentially eliminate certain suspects. In this study, we collected a total of 2 900 single digit fingerprints from 290 individuals, and the whorl axis slant was examined in the 743 whorl pattern fingerprints (385 from the right hand and 358 from the left hand). A slant towards the right side was present in 81.82% of samples from the right hand, while a slant towards the left side was observed in 80.73% of samples from the left hand. After applying a chi-square test to the dataset, the results were found to be statistically significant for the determination of hand origin. Our results suggest that the whorl axis slant in a fingerprint is indicative of hand origin (right or left).

**Key points**
- Single digit fingerprints with whorl pattern were analyzed.
- Whorl “axis slant” was used to determine the hand origin.
- Right axis slant would indicate the right hand of the print.
- Left axis slant would indicate the left hand of the print.

## Introduction

Fingerprints are the most common and valuable form of evidence for personal identification in forensic science. A fingerprint possesses a large number of specific characteristics that assist with identifying an individual. Extensive works are available describing fingerprint pattern distribution [1–8], fingerprint ridge count [9–12], ridge density [13–17], minutiae [5,6], and other dermatoglyphic features present on the fingertips [18,19]. In a typical forensic case, obtaining a full 10 digit set of fingerprints is highly unlikely. Forensic examiners are frequently left to work with only single digit fingerprint [20–22]. In such cases, appraising the hand becomes an important attribute that helps an investigating officer substantially reduce the suspect pool [21,22]. Nevertheless, very few studies are currently available that describe identification of the hand by single digit fingerprint [20–25].

Based on the available literature, the most commonly examined fingerprint pattern is the “whorl”. Some studies determined if the print belonged to the left or right hand based on six parameters (namely the slope of apex ridges, rotation of the central ridges, the angle formed at both sides of the core, position of the perpendicular bisector on the delta line, ridge tracing and ridge count) [23,24], while a later study added three parameters (viz. the angle between deltas and core, the direction of the pattern and distance between the deltas and the core) to the existing six [21]. One of the new parameters, precisely “the direction of the pattern” included the general sloping/inclination (upper portion) of the central pattern area of the whorl in the analysis [21]. A recent report proposed using the whorl axis slant (lower portion) to differentiate the origin of the prints from the left or right hand but was only done in Caucasian males [22]. It is essential to test this parameter in a larger sample as well as in both the sexes and in a mixed population for it to be applicable in a routine forensic scenario.

In this study, we aimed to determine the axis slant estimate right-hand or left-hand origin from whorl pattern in the heterogeneous population of central India.

## Materials and methods

### Criteria for participant selection

Healthy adult individuals between 18 and 60 years of age that did not belong to any homogenous population were randomly selected from Nagpur City, Maharashtra in central India for inclusion in this study. Any subject with a sign of significant injury or disease, especially related to the hand, were excluded from this study.

### Sample collection

A total of 2 900 single digit fingerprints were collected from 290 individuals (173 female and 117 male) with informed written consents, and the patterns were classified according to Henry's classification [4]. The present analysis was carried out on a total of 743 single digit whorl pattern fingerprints (385 of the right hand and 358 of the left hand). Fingerprints impressions were collected using the established method [4] (by first rolling the fingertips of both the hands (nail to nail) on a fingerprint plate smeared with black impression ink followed on the fingerprint slip) in the predefined format.

### Analysis parameters

**Figure 1.**
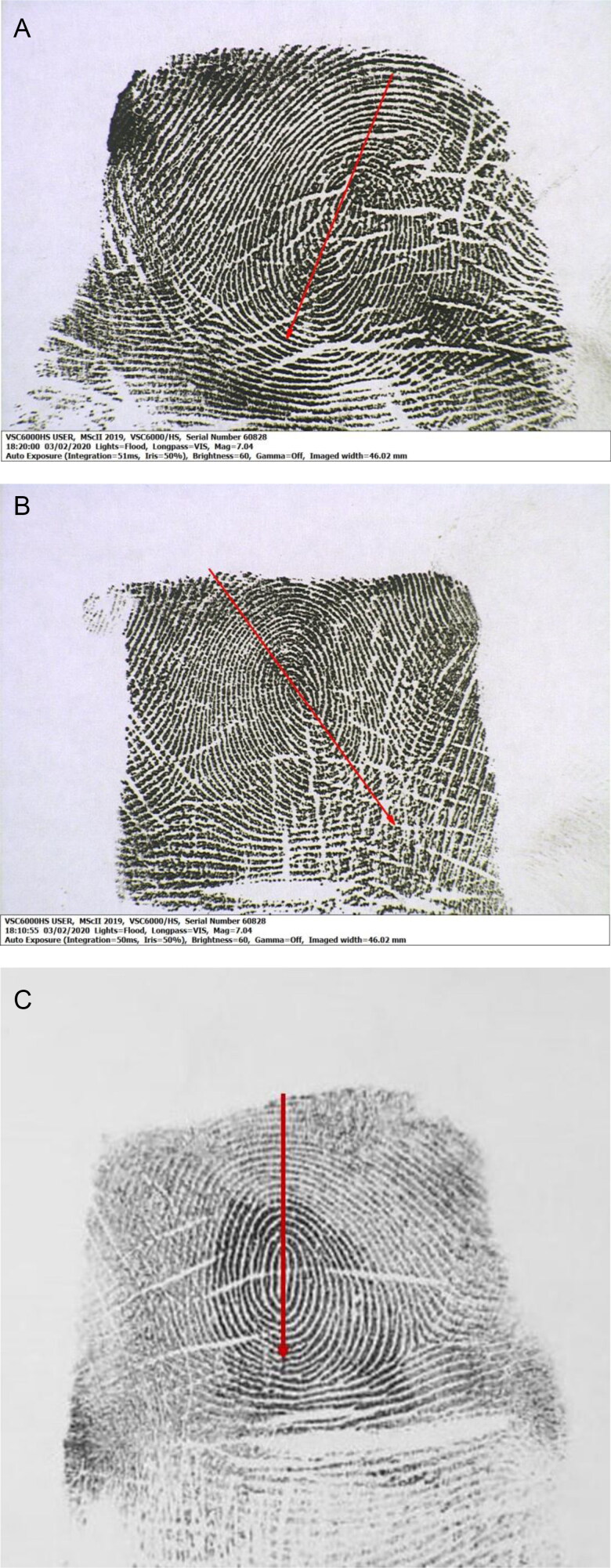
Right (A), left (B) and absent (C) whorl axis slants marked by the arrows (Image of the Fingerprint under Video Spectral Comparator).

The whorl axis is the imaginary line that runs from top to bottom in a whorl pattern and passes through the central pattern area in which it could be rotated. The “whorl axis slant” is defined as the direction in which the lower portion of this imaginary line is oriented in, and may be slanted towards either the right side or left side of the print [22]. If the lower portion of the axis is sloping towards the right, then it would be termed as a “right slant” (Figure 1A), while if it is sloping towards the left, it would be termed as a “left slant” (Figure 1B). If the whorl axis is sloping neither towards the right nor left, then it would be termed as “absent” to indicate the absence of a slant (Figure 1C).

In this study, the whorl axis slant in a total of 743 single digit whorls (385 of the right hand and 358 of the left hand) was scrutinized by two examiners (authors with 9 years of experience of teaching, research and forensic examination of fingerprints) using a handheld illuminated microscope and under video spectral comparator (VSC^®^6000/HS by Foster + Freeman) under “white floodlight” (Lights = Flood, Magnification = ×7.04, Auto Exposure). The “Draw Arrow” tool (of the inbuilt software VSC^®^ Suite) was used to draw coloured (red) arrows and make annotations on the images, as seen in the above-mentioned figures.

A blind test was also carried out. Thirty single digit whorl pattern fingerprints were randomly selected from the original 743 single digit whorls collected for analysis. These fingerprints were assessed by two observers, different from the two examiners mentioned previously (with primary education and training in fingerprint examination as a part of their Master's course curriculum), independently evaluated the axis slant in order to predict the hand of origin.

### Statistical analysis

Statistical analyses were performed using chi-square tests and *t*-tests, and *P* < 0.01 was considered to be significant.

## Results

Of the 2 900 fingerprints collected and classified, 743 (25.62%) were whorl patterns. For specific whorl subclassifications, 24.00% of the total fingerprints collected were spiral whorl (*n* = 696), while 1.62% were concentric whorl (*n* = 47). Of the whorl pattern fingerprints, 51.82% (*n* = 385) of them originated from the right hand, while 48.18% (*n* = 358) of them were from the left hand. Additionally, 55.05% (*n* = 409) of the whorl patterns came from females, and 44.95% (*n* = 334) came from males (Table 1). The overall fingerwise distribution of the whorl patterns in both hands in males and females is depicted in Supplementary Table S1.

**Table 1.** Occurrence of whorl pattern fingerprints in males and females (*n*, %).

Whorls	Male (*N* = 1 170)			Female (*N* = 1 730)			Grand total
	RH (585)	LH (585)	Total (1 170)	RH (865)	LH (865)	Total (1 730)	
Spiral whorl	159, 27.18	149, 25.47	308, 26.32	200, 23.12	188, 21.73	388, 22.43	696, 24.00
Concentric whorl	15, 2.56	11, 1.88	26, 2.22	11, 1.27	10, 1.16	21, 1.21	47, 1.62
Total	174, 29.74	160, 27.35	334, 28.55	211, 24.39	198, 22.89	409, 23.64	743, 25.62

RH: right hand; LH: left hand

**Table 2.** Comparison of the results of this study with those from studies in the literature.

Slant (%)	Present Study^a^		Brazelle & Brazelle [22]				Kapoor & Badiye [21]^a^	
			Examiner A^a^		Examiner B^a^			
	RH (*n* = 385)	LH (*n* = 358)	RH (*n* = 301)	LH (*n* = 249)	RH (*n* = 301)	LH (*n* = 249)	RH (*n* = 250)	LH (*n* = 250)
RS	81.82	9.22	85.05	6.83	88.04	8.43	79.2	14.4
LS	10.65	80.73	6.64	83.53	6.98	85.54	8.4	73.6
AB	7.53	10.05	8.31	9.64	4.98	6.02	12.4	12.0

RH: right hand; LH: left hand; RS: right side; LS: left side; AB: absent

^a^ *P* < 0.00001

In whorl fingerprints that originated from the right hand, 81.82% (*n* = 315) had a whorl axis slanted towards the right side, 10.65% (*n* = 41) slanted towards the left side, and 7.53% (*n* = 29) had no whorl axis slant present. Conversely, in whorl fingerprints that originated from the left hand, 80.73% (*n* = 289) exhibited a whorl axis slanted towards the left side, 9.22% (*n* = 33) slanted towards the right side, and 10.05% (*n* = 36) had no whorl axis slant present (Table 2).

**Table 3.** Blind trial study result by independent observers for determination of hand origin (right or left) (*n*, %).

Samples	Observer A	Observer B
Correct identification of hand	25, 83.33	24, 80.00
No identification of hand (due to absent whorl slant)	3, 10.00	3, 10.00
Incorrect identification of hand (due to opposite slant of whorl)	2, 6.67	2, 6.67
Incorrect identification of hand	0, –	1, 3.33
Total	30, 100	30, 100

After applying a chi-square test, the results were statistically significant (chi-square value = 415.214 and *P*-value <0.00001), suggesting that the whorl axis slant may be an indicator of the hand of origin. After applying a *t*-test, the inter- and intra-observer variabilities were found to be insignificant (*P* > 0.01), suggesting that there was no significant difference in the analysis results by the two examiners.

In the blind trial that was conducted with the whorl axis slants, the two observers were able to correctly identify the hand of origin for 25 (83.33%) and 24 (80.00%) samples, respectively out of the total 30 samples (Table 3), suggesting that even the apprentice observers were able to correctly use the parameter in consideration.

## Discussion

The present study was carried out on a more significant number of samples, including both the sexes and a heterogeneous population. The core findings of our study that the incidence of whorl axis slant towards the right side are more in the right hand whereas towards the left side in the left hand agree with the results published by Brazelle and Brazelle [22]. In their study, two fingerprint examiners independently analyzed the samples, and both reported a higher incidence of a right side slant in whorl patterns from the right hand and more left side slants in those from the left hand [22]. However, it is possible that the higher percentages observed may be attributed both to the studied population (i.e. Caucasians) and the inclusion of only males in their study (Table 2).

One parameter out of the nine investigated by Kapoor and Badiye [21] was the general sloping/inclination/tilting (of the upper portion) of the central main pattern area of the whorl. The lower portion of the main pattern area they studied is just opposite of the upper portion, i.e. if the inclination of the upper portion is towards the right then the slant of the lower portion would be towards the left and *vice versa*. Hence, those results would offer a better comparison if inversed [22]. This was determined to be the case based on our findings presented in this work. The slight differences in the number of “absents” that were observed may be attributed to the difference in the studied population and the increased sample size (Table 2). Data from all three studies showed the statistical significance of the whorl axis slant for hand origin determination but reported insignificant inter- and intra-observer variations. This suggests that the parameter were fairly accurate even when used repeatedly by the same observer and by different observers.

In the blind test trial (Table 3), the prints that could not be identified by the observers included three prints each with an “absent” axis slant. The (same) two samples were incorrectly identified by both the observers because of an oppositely oriented slant in these prints, meaning a right slant in the left hand and a left slant in the right hand. One print was incorrectly identified by one of the two observers, suggesting that human error due to inexperience may affect the results.

## Conclusion

Taken together, the results of this study suggest that right slanted and left slanted whorl axes imply that the fingerprints originated from the right and left hands, respectively. Our results confirmed the findings of earlier studies regarding the utility of the whorl axis slant for the determination of hand origin. However, caution must be exercised when trying to draw conclusions based on a single parameter. Examining a combination of parameters may likely be useful for improving the probability of correct determination of hand origin. Use of the whorl axis slant as a tool for fingerprint comparisons could be incorporated in the ACE-V (Analysis, Comparison, Evaluation and Verification) process, possibly resulting in a more comprehensive, efficient, and rapid analysis. Investigations on other fingerprint patterns and the use of additional parameters for the determination of hand origin are currently in progress.

Further studies are needed to evaluate the effect of population variation on results. Likewise, non-homogenous population samples must also be considered. With increased testing, efficacy, proficiency and speed, the determination of hand origin (right/left) would undoubtedly be helpful in fingerprint examination.

## Supplementary Material

Supplemental Material
