# Supplementary material for: Fingerprint analysis for the determination of hand origin (right/left) using the axis slant in whorl patterns
Source: Forensic Sci Res. 2020 Aug 12;7(2):285–9. doi: 10.1080/20961790.2020.1794362 (PMC9245989; doi:10.1080/20961790.2020.1794362)
Supplement: Supplemental Material [file TFSR_A_1794362_SM6207.docx]

**Supplementary Table S1. Fingerwise distribution of whorl pattern fingerprints from both hands in males and females**

| Patterns | Right hand | | | | | | | | | | | | Left hand | | | | | | | | | | | | Both hands | |
| --- | --- | --- | --- | --- | --- | --- | --- | --- | --- | --- | --- | --- | --- | --- | --- | --- | --- | --- | --- | --- | --- | --- | --- | --- | --- | --- |
|  | Thumb | | Index | | Middle | | Ring | | Little | | Right Total | | Thumb | | Index | | Middle | | Ring | | Little | | Left Total | |  |  |
|  | *n* | % | *n* | % | *n* | % | *n* | % | *n* | % | *n* | % | *n* | % | *n* | % | *n* | % | *n* | % | *n* | % | *n* | % | *N* | % |
| SW | 69 | 9.29 | 71 | 9.56 | 45 | 6.06 | 127 | 17.09 | 47 | 6.33 | 359 | 48.32 | 52 | 7.00 | 85 | 11.44 | 43 | 5.79 | 112 | 15.07 | 45 | 6.06 | 337 | 45.36 | 696 | 93.67 |
| CW | 10 | 1.35 | 7 | 0.94 | 2 | 0.27 | 7 | 0.94 | 0 | 0.00 | 26 | 3.50 | 4 | 0.54 | 7 | 0.94 | 3 | 0.40 | 7 | 0.94 | 0 | 0.00 | 21 | 2.83 | 47 | 6.33 |
| Total | 79 | 10.63 | 78 | 10.50 | 47 | 6.33 | 134 | 18.03 | 47 | 6.33 | 385 | 51.82 | 56 | 7.54 | 92 | 12.38 | 46 | 6.19 | 119 | 16.02 | 45 | 6.06 | 358 | 48.18 | 743 | 100.00 |

SW: spiral whorl; CW: concentric whorl
